# High-Grade Malignant Peripheral Nerve Sheath Tumor Arising From Common Peroneal Nerve Neurofibroma

**DOI:** 10.7759/cureus.59607

**Published:** 2024-05-03

**Authors:** Jian-Jiun Chen, Chien-Kuan Lee, Chen-Yuan Yang

**Affiliations:** 1 Department of Orthopedics and Traumatology, Taipei Veterans General Hospital, Taipei, TWN; 2 Department of Pathology, Kuang Tien General Hospital, Taichung, TWN; 3 Department of Orthopedic Surgery, Kuang Tien General Hospital, Taichung, TWN

**Keywords:** popliteal mass, common peroneal nerve, malignant peripheral nerve sheath tumor, neurofibroma, neurofibromatosis

## Abstract

This article presents a case report of a 45-year-old male with neurofibromatosis type I (NF1) who developed a high-grade malignant peripheral nerve sheath tumor (MPNST) originating from a neurofibroma within the common peroneal nerve over popliteal fossa. MPNSTs are aggressive tumors associated with NF1, causing significant mortality. The patient underwent tumor resection surgery and received postoperative radiation therapy. Follow-up examinations showed no impairment of motor function and no tumor recurrence after regular MRI evaluation for four years. This article explores the challenges of distinguishing benign neurofibromas from malignant MPNST via MRI image and biopsy, and achieving a balance between tumor excision and preserving nerve functionality during surgical treatment. However, caution is warranted due to the risk of recurrence.

## Introduction

Neurofibroma stands as the most prevalent peripheral neurogenic neoplasm in individuals diagnosed with neurofibromatosis type I (NF1). This benign neoplastic growth originates from the nerve sheath and may manifest as a solitary mass or as multiple masses within the confines of any nerve distributed throughout the body. The clinical manifestations of neurofibroma exhibit variability contingent upon the specific anatomical site and the specific type of lesion [1].

Although neurofibromas are typically benign, there is a possibility for them to undergo transformation into aggressive malignant peripheral nerve sheath tumors (MPNSTs). MPNSTs are distinguished by their aggressive nature, and these tumors are recognized for their correlation with unfavorable survival rates. Indeed, they stand as the primary contributor to mortality among individuals diagnosed with NF1 [2].

Regardless of whether they are benign or malignant, neurofibromas pose a challenge during surgery as they cannot be easily distinguished from normal nerve fascicles. Currently, the main treatment approach for MPNST is wide excision, which involves the complete removal of the tumor along with a margin of healthy tissue. However, this surgical method often results in sacrificing a significant amount of nerve functionality [3, 4].

This case report presents a patient with neurofibromatosis type I (NF1) who developed a rare high-grade MPNST within the common peroneal nerve over the popliteal fossa. The patient underwent a tumor resection surgery and postoperative radiation therapy. After follow-up for four years, there were no signs of tumor recurrence, and the patient did not experience any neurologic deficits.

## Case presentation

Patient history

The patient is a 45-year-old male with a medical history of NF1. He has been experiencing a palpable firm mass lesion in the right popliteal fossa for decades. Although he does not have motor weakness, he does report intermittent tingling sensations in the dorsal foot. Additionally, he has been suffering from intractable tumor pain that worsened rapidly over the past three months, to the extent that he is unable to fully extend his knee joint even when lying down, and he had to sleep in the sitting position. A biopsy of the mass was performed elsewhere 14 years ago with a diagnosis of neurofibroma. His daughter also has NF1, evidenced by the presence of café au lait spots on her body and congenital pseudoarthrosis of the tibia undergoing multiple reconstruction surgeries.

Investigation

**Figure 1:**
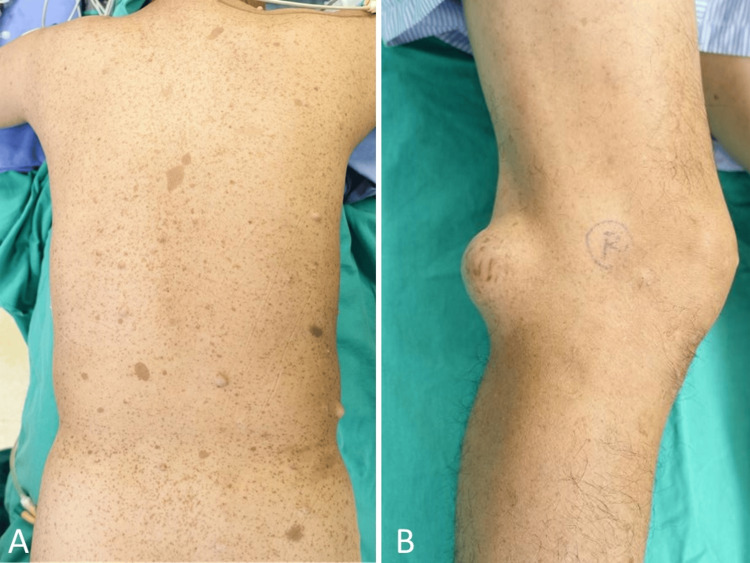
Cafe au lait spots and popliteal fossa mass A) Numerous cafe au lait spots on the patient's body; B) 7cm bulging firm mass located in the right knee posterolateral area of the popliteal fossa

Upon physical examination, the patient exhibited numerous cafe au lait spots on the body (Figure 1) and had a 7cm bulging firm mass located in the right popliteal fossa. There were no signs of numbness or motor weakness. Tinel's sign was positive.

**Figure 2:**
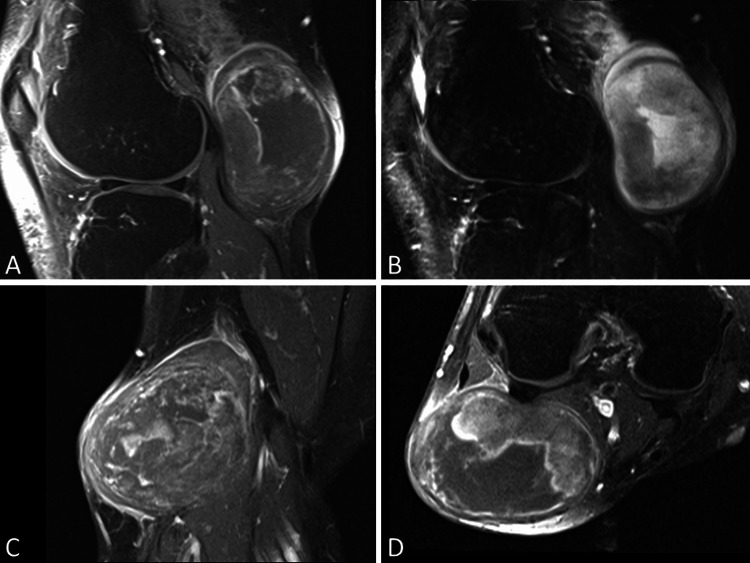
MRI characterization of the tumor within the peroneal nerve MRI revealed a 7 cm well-defined mass lesion within the peroneal nerve. The lesion exhibited mildly low signal intensity in the T1 sequence with contrast enhancement (A) and high signal intensity with peripheral edema in the T2 sequence (B). The coronal (C) and axial (D) view showed core region heterogeneity of the mass.

The X-ray images of the right knee in both the anterior-posterior and lateral views revealed no calcification but a soft tissue opacity in the posterolateral region of the knee joint. Subsequent magnetic resonance imaging (MRI) with contrast identified a 7cm well-encapsulated mass located within the peroneal nerve and demonstrated a core region with heterogeneous signal intensity, surrounded by peripheral edema (Figure 2).

Differential diagnosis

Considering the patient's age and the lack of trauma or pre-existing joint pathology, the likelihood of a secondary Baker's cyst was diminished. The clinical evaluation, along with preoperative imaging, aided in excluding potential diagnoses such as sebaceous cyst, lipoma, hemangioma, muscular swelling, or osseous pathology. 

Although the preoperative MRI image indicates a core region with heterogeneous signal intensity, surrounded by peripheral edema, suggesting that it is a potential non-benign tumor, the preoperative diagnosis still prioritized the suspicion of neurofibroma over MPNST. This decision is primarily based on the established diagnosis and family history of neurofibromatosis type 1, along with a previous pathological report from another hospital's biopsy confirming neurofibroma. We overlooked the substantial size and prolonged duration of this mass, which increases the risk of malignant transformation.

Treatment

**Figure 3:**
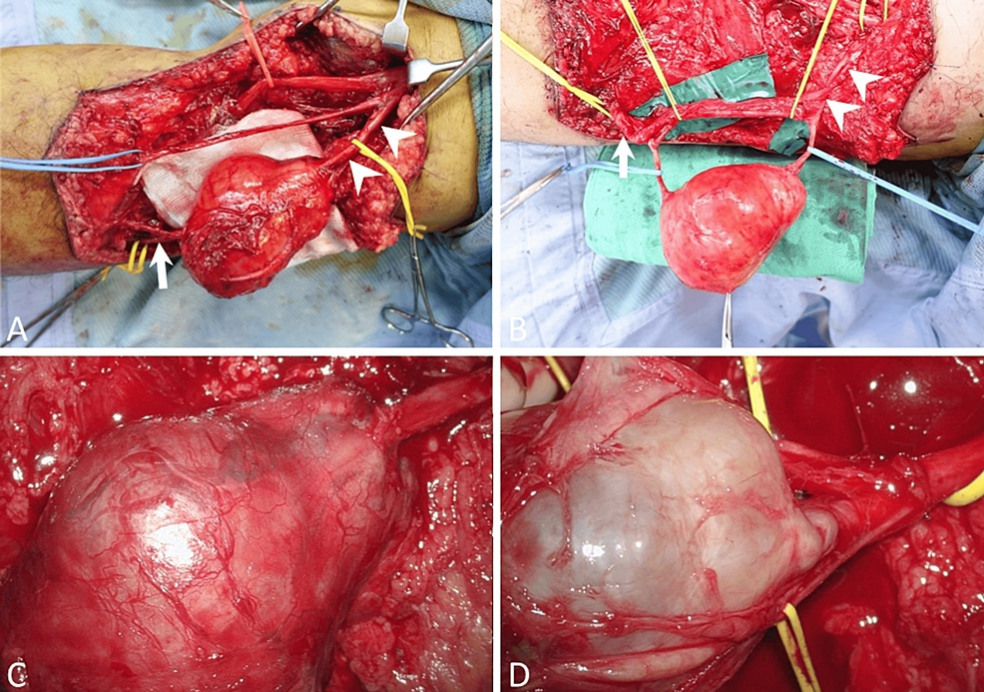
Gross and microsurgical dissection of MPNST in common peroneal nerve The tumor was located between the proximal end (arrowhead) and the distal end (arrow) of the common peroneal nerve tagged with yellow vessel loops (A). After dissection, the MPNST within the nerve fascicle was isolated from the main trunk of the common peroneal nerve (B); The tumor before (C) and after (D) intrafascicular tumor dissection under the microscope. MPNST - malignant peripheral nerve sheath tumor

Due to the location of the tumor within the common peroneal nerve, preserving the integrity of the nerve was of utmost importance to minimize the impact on the patient's postoperative functional activities. Therefore, we opted for an intrafascicular tumor resection assisted by a microscope. Under prone position and posterior lazy S incision, gross dissection and identification of the tibial, sural, and common peroneal nerves were performed first (Figure 3). Then, meticulous dissection within the peroneal nerve fascicles was done under a microscope, and the neurofibroma within the nerve fascicle was isolated. To assess nerve function, electric stimulation was applied to the fascicles of the common peroneal nerve, and fascicle function was assumed normal with reacting ankle dorsiflexion. The neurofibroma within the solitary nerve fascicle was excised, and the remaining nerve stumps were primarily repaired. Following the surgery, adjuvant radiotherapy (60 Gy in 30 fractions) was administered.

Outcome and follow-up

**Figure 4:**
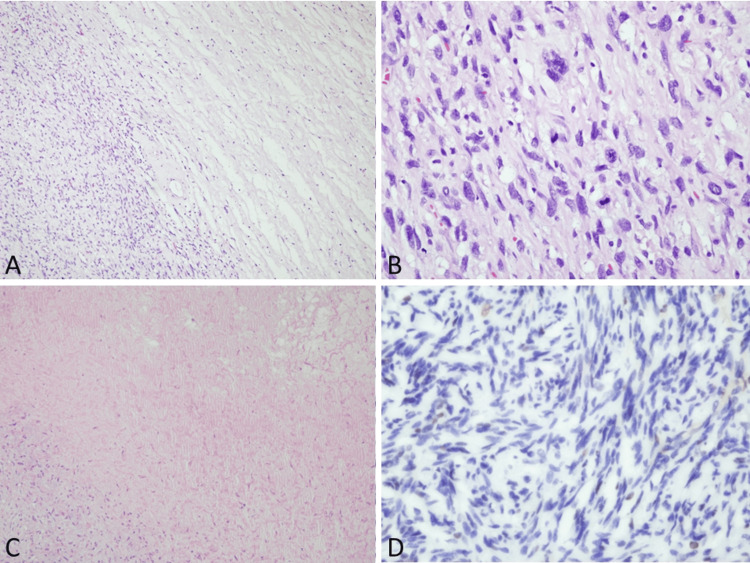
Histopathological and immunohistochemical findings of MPNST A) High-grade tumor with hypercellularity in the left side and usual neurofibroma in the right side (40X); B) High-grade MPNST showing nuclear pleomorphism, hyperchromatic nuclei, and increased mitosis(400X); C) Tumor necrosis in MPNST (400X); D) Loss of H3K27me3 in immunohistochemical staining (400X) MPNST - malignant peripheral nerve sheath tumor

The pathological diagnosis confirmed a high-grade malignant peripheral nerve sheath tumor originating from a neurofibroma (Figure 4). Microscopic examination revealed the tumor size to be 6.8 x 6.0 x 2.3 cm, with a high mitotic rate of 9/10 high-power fields (HPF) and necrosis. The surgical margins were clear of malignant tumor cells but involved neurofibromatosis. Ancillary studies indicated positive immunohistochemical stains for S100 protein and Ki67 (20%), while H3K27me3 showed equivocal and heterogeneously lost results. Subsequent examinations included a whole bone scan and chest CT. The whole-body bone scan showed no evidence of bone metastasis, while the chest CT revealed no lung metastasis. However, bilateral hypovascular lesions were detected in the upper mediastinum, raising suspicion of bilateral vagal nerve neurofibromas. Since the patient did not experience any symptoms related to it, no further surgery was performed. Following the operation, the patient experienced mild numbness in the dorsal foot and lateral calf, along with slight weakness in the ankle and toe dorsiflexion. His motor function gradually improved, and he regained the ability to walk without assistance four weeks after the operation. Strength assessment was conducted using the Medical Research Council (MRC) grade, with immediate postoperative M3, postoperative two weeks M4, and postoperative four weeks M5. After four years of regular follow-up, there were no signs of local recurrence by physical examination and MRI evaluation. 

## Discussion

MPNST accounts for approximately 10% of the entirety of soft tissue sarcomas, with about 8-13% of patients with NF1 being affected by this aggressive tumor [2,5]. Nearly 50% of all MPNST cases occur in individuals with NF1 mutations. The residual cases comprise 45% of sporadic MPNST cases with unknown genetic anomalies, while the remainder correlates with prior radiotherapy exposure. In NF1-associated MPNST, the progression from pNF to MPNST is challenging to identify using radiological imaging or biopsy. This difficulty is attributed to the intralesional heterogeneity exhibited by these tumors [6].

MRI is commonly employed to identify the location and assess the size and invasiveness of neurofibroma tumors. It enhances the contrast between the tumor and surrounding tissues. However, MRI and/or computerized tomography (CT) imaging cannot consistently determine whether the tumor has undergone malignant transformation [7,8]. In contrast, positron emission tomography using the glucose analog 18 fluorodeoxyglucose (FDG-PET) can discover elevated metabolic activity in malignant tumors, enabling successful differentiation between MPNST and pNF. FDG-PET also enables the assessment of the malignant tumor's grade within a heterogeneous lesion [8]. Presently, quantitative FDG-PET imaging with either CT or MRI provides the most accurate means of distinguishing between benign tumors and MPNST [9,10]. Although FDG-PET is beneficial in differential diagnosis, Taiwan's national health insurance regulations do not include MPNST as an approved indication. In this situation, the patient expressed an inability to afford the medical expenses for this diagnostic procedure.

In terms of histopathological features, the identification of perivascular hypercellularity, tumor herniation into vascular lumens, and necrosis collectively serve as indicators for the presence of MPNST [11]. Regarding immunohistochemical features, complete loss of SOX10, neurofibromin, or p16 expression, as well as the manifestation of estimated glomerular filtration rate (eGFR) immunoreactivity, specifically points to MPNST. In addition, loss of staining for H3K27me3 may be helpful in the diagnosis of MPNST [12]. Ki-67 labeling indices ≥20% have demonstrated a high predictive value for MPNST, with a sensitivity of 87% and specificity of 96% [11].

Currently, there is no clear consensus on the differentiation between low-grade and high-grade MPNST. However, research indicates that pathologic diagnosis can distinguish between the two. Low-grade features include cytologic atypia, loss of neurofibroma architecture, hypercellularity, a mitotic index of 3-9/10 HPFs, and the absence of necrosis. On the other hand, high-grade MPNST is characterized by a mitotic rate of at least 10/10 HPFs or 3-9/10 HPFs combined with necrosis [13]. In our patient, the mitotic rate was 9/10 HPF, and the presence of necrosis was noted. Therefore, the final diagnosis was classified as high-grade MPNST.

As for the surgical treatment in this case, which involves a large malignant nerve tumor occupying the popliteal fossa, we agreed that choosing a wide resection, instead of intrafascicular resection, is a safe, rational, and standard procedure [14,15]. However, it may not be the optimal choice for this patient. Postoperatively, we had discussed with the patient, explaining that opting for wide resection would entail a more comprehensive removal of the tumor, encompassing the entire segment of the common peroneal nerve and surrounding normal tissues. This approach aims to reduce the rates of recurrence and distant metastasis. Putting aside the challenges, complications, and medical costs associated with nerve reconstruction, bridging such a substantial nerve gap (>7cm) with either an autogenous sural nerve graft or an allogenous nerve graft implies that the patient would undergo an extended period of nerve regeneration and rehabilitation, lasting for several years, and final functional recovery is often less satisfactory [3,16]. For a patient who had normal preoperative functionality, he expressed an inability to accept such an outcome under any circumstances. Therefore, if the preoperative diagnosis had confirmed MPNST, we would have engaged in thorough communication with the patient regarding the surgical indications, prognosis, and potential complications. For this reported case, we would respect the patient's choice and may not opt for wide resection as the initial approach. Instead, intrafascicular tumor resection and adjuvant radiotherapy would still be considered to preserve the patient's nerve. Fortunately, the patient has been recurrence-free for a complete four-year follow-up, with normal functionality.

Regarding adjuvant therapy, adjuvant radiotherapy is recommended for high-grade tumors (>5 cm) in cases where complete resection cannot be achieved [16,17]. Additionally, literature suggests that the use of 95% alcohol or liquid nitrogen spray has been shown to effectively reduce the recurrence rate of tumors. The nerve damage caused when the fascicle remains intact has the potential for self-recovery, albeit with a longer duration [18,19]. However, we still have concerns about the potential for irreversible nerve damage with the use of 95% alcohol. Furthermore, we believe the use of liquid nitrogen spray should be considered as a suitable adjuvant procedure if available in our hospital.

MPNSTs exhibit unfavorable survival outcomes, serving as the predominant contributor to mortality among individuals with NF1 [2]. Current research has reported five-year progression-free survival rates of 18% and five-year disease-specific survival rates of 32% for MPNSTs [11]. Numerous poor prognosis factors have been identified, including tumor size greater than 5 cm, local recurrence, high-grade morphology, and tumors located in the trunk [20]. Our patient meets the criteria of high grade and a size larger than 5 cm; therefore, after the surgery, we closely monitored the patient for signs of recurrence or metastasis. As of the four-year postoperative follow-up, the patient has not exhibited any neurological symptoms or palpable masses, and an MRI revealed no signs of local recurrence. 

## Conclusions

If NF1 presents with a peripheral neurogenic tumor larger than 5 cm, and MRI reveals core heterogeneity with peripheral edema, the possibility of MPNST should be considered, even if a prior biopsy result was benign. This case report demonstrates an NF1 patient with MPNST arising from common peroneal nerve neurofibroma in the popliteal area. Following marginal excision and radiotherapy, the patient has remained free of recurrence for four years. Careful monitoring is still warranted due to the high risk of recurrence.
